# Selective Quenching of Peracetic Acid by Sodium Dithionite Enables Rapid, Non-Thermal Sterilization for *Euglena gracilis* Cultivation

**DOI:** 10.3390/microorganisms14020315

**Published:** 2026-01-29

**Authors:** Hyun-Jin Lim, Min-Su Kang, Min-Sung Kim, Jong-Hee Kwon

**Affiliations:** 1 Division of Applied Life Sciences (BK21), Gyeongsang National University, Jinju 52828, Republic of Korea; 2 Department of Food Science & Technology, Institute of Agriculture & Life Science, Gyeongsang National University, Jinju 52828, Republic of Korea

**Keywords:** sterilization, peracetic acid, sodium dithionite, *Euglena*, paramylon

## Abstract

Peracetic acid (PAA) has strong biocidal activity against bacteria, fungi, and spores, even with short contact times. PAA-mediated sterilization is therefore an attractive method for sterilization of growth media that have heat-labile components or when polymer-based equipment is used. However, residual PAA and co-existing hydrogen peroxide (H_2_O_2_) can inhibit the growth of cultivated species, necessitating a fast and reliable quenching strategy that does not require rinsing. In contrast to Fe–EDTA-based catalytic decomposition that is strongly influenced by pH, buffers, and organic nitrogen, we demonstrate a fundamentally different, stoichiometric quenching strategy using sodium dithionite that enables instantaneous and selective removal of PAA. Na_2_S_2_O_4_ preferentially reduced PAA over H_2_O_2_ in a 0.03% PAA solution and achieved complete PAA reduction within 5 s, independent of pH and in the presence of nitrogen compounds. By adjusting the Na_2_S_2_O_4_ dose, PAA could be selectively removed while allowing a small fraction of H_2_O_2_ to remain. When applied to the cultivation of *Euglena gracilis*, which tolerates low levels of H_2_O_2_, the PAA–Na_2_S_2_O_4_-treated medium resulted in greater cell growth and higher paramylon production than autoclaved medium.

## 1. Introduction

*Euglena* is a genus of unicellular, photosynthetic, flagellated eukaryotes that mostly inhabit freshwater environments. *E. gracilis* can produce a variety of high-value compounds, such as paramylon starch, making it potentially valuable as a sustainable next-generation biotechnology platform [1,2]. In addition, biomass from *E. gracilis* can be used as a source of digestible proteins, unsaturated fatty acids, vitamins, and antioxidant compounds for the food and pharmaceutical industries [3,4,5,6,7]. *Euglena*-based systems can also couple CO_2_ fixation with nutrient removal from wastewater as part of a resource-circulating process and have potential use for the environmentally friendly production of biodiesel and the sustainable production of aviation fuel precursors [8,9].

The stable and reproducible manufacture of these high-value products requires axenic cultivation to prevent nutrient competition and metabolic interference by contaminating microorganisms [10]. Although autoclaving at high temperature and high-pressure is a common method of sterilization, it cannot be used with growth media and equipment that is heat-sensitive and it requires substantial energy when performed at a large scale [11,12,13]. Chemical sterilization is an important alternative, because it can be applied to heat-sensitive media and equipment [14,15,16,17,18,19]. Chemical sterilization commonly uses agents such as H_2_O_2_, NaClO, Ca(ClO)_2_, and peracetic acid (PAA), but these agents require careful handling and proper disposal and frequently necessitate labor-intensive rinsing with sterilized water to remove residual disinfectant prior to cell inoculation [14,20,21,22].

To address these issues, recent approaches have moved toward integrated chemical sterilization–neutralization strategies that enable concurrent sterilization of the culture medium and cultivation system. PAA has strong and broad-spectrum antimicrobial activity against bacteria, fungi, spores, and even some biofilms after short contact times [15,16,18,19,23], and it can be decomposed and neutralized via ferric ion (Fe^3+^)-mediated reactions. Building on this principle, previous researchers who used a polymer-based bioreactor and glucose-containing medium performed simultaneous sterilization with PAA and neutralization via Fe-EDTA-mediated PAA decomposition/neutralization (hereafter, Fe-EDTA-mediated decomposition) in HEPES buffer [15,16,18,19]. This integrated sterilization–neutralization approach enabled mono-cultivation without washing or filtration and led to a higher biomass yield than conventional thermal sterilization. However, Fe–EDTA mediated PAA decomposition relies on metal-catalyzed Fe(III)/Fe(II) redox cycling, which requires tightly controlled conditions such as near-neutral pH, buffering, and stable iron speciation. In practical cultivation media, however, these requirements are difficult to satisfy due to complex organic and inorganic constituents that perturb iron chemistry, making the process highly sensitive to medium composition and limiting its applicability across diverse microorganisms. Sodium dithionite (Na_2_S_2_O_4_) exists as the dithionite ion (S_2_O_4_^2−^) in aqueous solution and functions as a strong and rapid reducing agent [24,25]. Under diverse conditions, dithionite can generate highly reactive species that rapidly reduce and deactivate oxidizing compounds, such as PAA and H_2_O_2_.

The purpose of this study of *E. gracilis* was to determine the optimal dose of Na_2_S_2_O_4_ needed to rapidly and completely neutralize PAA-sterilized media without using Fe-EDTA-mediated decomposition or any additional reactions. We also examined the effect of medium conditions (pH, HEPES buffer, EDTA, and organic nitrogen) on the rapid Na_2_S_2_O_4_-mediated decomposition of PAA. We hypothesize that the differential reactivity of Na_2_S_2_O_4_ toward PAA and H_2_O_2_ and *Euglena*’s high tolerance to H_2_O_2_ will enable selective quenching of PAA at a moderate concentration of Na_2_S_2_O_4_, so that any residual Na_2_S_2_O_4_ does not inhibit *Euglena* growth. We also determined whether any reaction byproducts that formed during the PAA-Na_2_S_2_O_4_ neutralization reaction affected cell growth and paramylon production relative to control cells that were grown in autoclaved medium.

## 2. Materials and Methods

### 2.1. Chemicals

PAA (20%) was from Dong Myung ONC Corp. (Busan, Republic of Korea) and sodium dithionite (83.0%) was from Daejung Chemicals & Metals Co., Ltd. (Siheung, Republic of Korea). Working solutions were prepared using triple-distilled water. Chemicals used in the growth medium are described below.

### 2.2. Instrumentation

Fixed-wavelength absorption measurements at 400, 515, and 680 nm were acquired using a UV-Vis spectrophotometer (V-760, JASCO, Tokyo, Japan) with a 10 mm cell. The pH was measured using a digital pH meter (Mettler-Toledo InLab Ultra Micro-ISM, Greifensee, Switzerland). A colorimetric test strip (Peroxide Test Indicator, Merck, Darmstadt, Germany) was used to verify the decomposition of PAA by Na_2_S_2_O_4_.

### 2.3. Colorimetric Determination of PAA and H_2_O_2_

PAA solutions are typically present as an equilibrium mixture of PAA (CH_3_COOOH), hydrogen peroxide (H_2_O_2_), acetic acid (CH_3_COOH), and water. The concentrations of PAA and H_2_O_2_ in PAA solutions were determined by a DPD/iodide colorimetric assay and a TiO-Ox colorimetric assay [26], respectively. Based on the commonly reported performance of the DPD/iodide colorimetric assay, the LOQ (limit of quantification) was approximately 0.00000025% (*w*/*v*) for PAA. Likewise, for the TiO-Ox colorimetric assay used for H_2_O_2_ determination, the LOQ was approximately 0.00000127% (*w*/*v*) [26].

### 2.4. Effect of Glucose on Degradation of PAA and H_2_O_2_ by Fe-Mediated Catalysis

Our previous study described the Fe-EDTA-mediated decomposition of PAA, in which HEPES was required to maintain pH and ensure complete degradation of peroxide species [19]. Thus, the effect of glucose on the complete decomposition of peroxide in the absence of HEPES was examined herein. A 0.03% PAA solution was prepared by diluting a 20% PAA stock solution with distilled water. A FeCl_3_-EDTA stock solution (96 mM FeCl_3_ and 96 mM EDTA) was then added to the 0.03% PAA solution that contained 0 or 20 g/L glucose to achieve a final FeCl_3_-EDTA concentration of 96 μM. The pH was then adjusted to approximately 7.0 using 5 M NaOH. Then, changes in the concentrations of PAA and H_2_O_2_ in the 0.03% PAA solution were monitored for 48 h using redox reaction-based colorimetric methods [26].

### 2.5. Effect of Peptone on Fe-EDTA-Mediated Degradation of PAA and H_2_O_2_

The Fe-EDTA-mediated decomposition of PAA can be inhibited by organic nitrogen compounds, such as peptone. To determine the effect of peptone on PAA decomposition in the presence of glucose, 5 g/L peptone was added to the 0.03% PAA solution that contained 20 g/L glucose. During the Fe-catalyzed conditions, changes in the concentrations of PAA and H_2_O_2_ were monitored for 48 h using colorimetric methods [26].

### 2.6. Decomposition of PAA by Reaction with Na_2_S_2_O_4_

A PAA solution contains PAA and H_2_O_2_, and Na_2_S_2_O_4_ reduces the PAA to acetic acid and the H_2_O_2_ to water. To determine the Na_2_S_2_O_4_ concentration required to completely reduce PAA and H_2_O_2_ in a 0.03% PAA solution, a 0.8 M Na_2_S_2_O_4_ stock solution was added to a 0.03% PAA solution to achieve a final Na_2_S_2_O_4_ concentration of 2.4, 4.8, 7.2, 9.6, or 12.0 mM (The Na_2_S_2_O_4_ concentration that is stoichiometrically equivalent to 0.03% PAA is approximately 4 mM). Then, residual PAA and H_2_O_2_ were quantified using colorimetric methods [26].

### 2.7. Rapid Na_2_S_2_O_4_-Mediated Decomposition of PAA Under Different Medium Conditions (pH, HEPES Buffer, EDTA, and Organic Nitrogen)

When a solution contains organic nitrogen, the Fe-EDTA-mediated decomposition of PAA is delayed, regardless of the presence of glucose or HEPES [16]. Therefore, the rapid Na_2_S_2_O_4_-mediated quenching of PAA was practically examined under *Euglena* growth medium conditions (see Section 2.9) in the presence of peptone. Briefly, *Euglena* growth medium containing 5 g/L peptone was sterilized with 0.03% PAA and subsequently treated with 9.6 mM Na_2_S_2_O_4_ to confirm complete neutralization of all PAA-derived peroxides. The presence of residual peroxides was assessed after 5 s using a Peroxide Test Indicator (Merck). In addition, we evaluated whether Na_2_S_2_O_4_ could completely quench a 0.03% PAA solution under various pH conditions (3, 5, 7, 9, and 11) and the presence of HEPES buffer or EDTA in *Euglena* medium.

### 2.8. Tolerance of Cells to Residual Na_2_S_2_O_4_

To evaluate the concentration-dependent cytotoxicity of Na_2_S_2_O_4_, a 0.8 M Na_2_S_2_O_4_ stock solution was added to actively motile cultures of *E. gracilis* to achieve final Na_2_S_2_O_4_ concentrations of 0.008, 0.016, 0.024, 0.032, or 0.040 mM. Changes in cellular activity were assessed after 5 min.

### 2.9. Cultivation of Cells

*E. gracilis* UTEX367 was purchased from the UTEX Culture Collection of Algae (Austin, TX, USA). The growth medium consisted of (g/L) NH_4_Cl—1, MgSO_4_·7H_2_O—0.1, CaCl_2_·2H_2_O—0.05, NaH_2_PO_4_—0.05, K_2_HPO_4_—0.05, Na_2_EDTA·2H_2_O—0.05, ZnSO_4_·7H_2_O—0.022, H_3_BO_3_—0.011, MnCl_2_·4H_2_O—0.005, FeSO_4_·7H_2_O—0.005, CoCl_2_·6H_2_O—0.0016, CuSO_4_·5H_2_O—0.0016, and (NH_4_)_6_Mo_7_O_24_·4H_2_O—0.0011. The growth factors (vitamins) were added at the following concentrations (mg/L): thiamine—0.01, biotin—0.002, cyanocobalamin—0.001, and pyridoxine—0.001.

Sodium acetate (10 g/L) was the primary source of organic carbon. Four cultivation conditions were established based on the presence of acetate (+Ac, −Ac) and the type of sterilization (PAA, autoclaving): (i) +Ac/PAA-sterilized, (ii) −Ac/PAA-sterilized, (iii) +Ac/autoclaved, and (iv) −Ac/autoclaved. For PAA sterilization, the PAA concentration was adjusted to 0.03%, and the medium was added to 500 mL T-flasks (GVS, Bologna, Italy) for 24 h. PAA was then completely quenched by adding Na_2_S_2_O_4_ (final concentration 7.2 mM), followed by adjustment of pH to approximately 7.0 using 5 M NaOH to provide pH conditions favorable for *Euglena* growth, and then inoculation with *E. gracilis*. The autoclaved control consisted of sterile growth medium that was autoclaved at 121 °C for at least 20 min. Cells were grown in the 500 mL T-flasks under agitation at 70 rpm and 25 °C, with continuous illumination from a flat LED panel that had a photosynthetic photon flux density of 50 µmol photons m^−2^ s^−1^. The growth of cells was monitored at 2-day intervals by measurement of OD_680nm_ using a UV-Vis spectrophotometer spectrophotometer (V-760, JASCO, Tokyo, Japan) with a 10 mm glass cuvette. Each result was expressed as the mean and standard deviation of four replicates.

### 2.10. Determination of Paramylon

The paramylon content was measured using a gravimetric method [27]. First, 50 mg of a freeze-dried sample of cells was suspended in 5 mL of acetone to remove the chlorophyll, and the cells were then lysed by sonication for 30 min. The sample was then suspended in acetone and sonicated as above, centrifuged at 4000 rpm for 5 min, suspended in 1% sodium dodecyl sulfate (SDS) to remove substances other than paramylon, and boiled at 100 °C for 30 min. The samples were then cooled to room temperature, boiled in SDS a second time, and then washed twice in distilled water. Finally, the sample was transferred to a 1.5 mL microtube and centrifuged at 4000 rpm for 5 min. The supernatant was discarded, and the pellet was dried overnight at 80 °C and then weighed. The paramylon content (%) was expressed relative to dry cell weight. Each result was expressed as the mean and standard deviation of three replicates.

### 2.11. Statistical Analysis

Data were analyzed using Statistical Analysis System software SAS version 9.4 for Windows (SAS Institute, Inc., Cary, NC, USA). Group differences were determined by a one-way ANOVA followed by Duncan’s test. A *p*-value below 0.05 was considered significant.

## 3. Results

### 3.1. Glucose-Enhanced Fe-Catalyzed Decomposition of PAA

**Figure 1:**
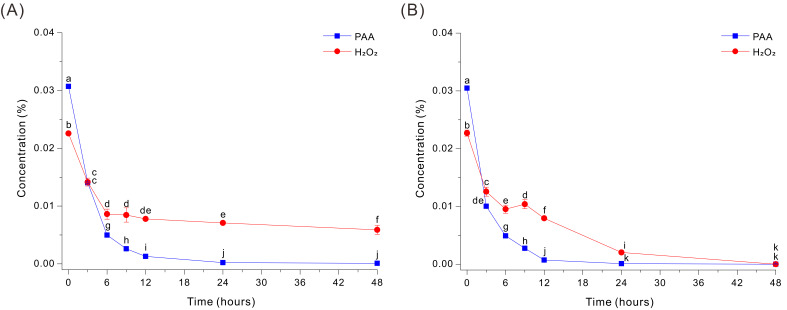
Decomposition of PAA and H_2_O_2_ by a Fe-EDTA-mediated reaction in a HEPES-free 0.03% PAA solution without glucose (**A**) or with 20 g/L glucose (**B**). The data were analyzed using one-way ANOVA, followed by Duncan’s test (*p* < 0.05). Lowercase letters indicate statistically significant differences among groups.

In the absence of HEPES, the Fe-EDTA-mediated decomposition of PAA in the 0.03% PAA solution at pH 7 was very rapid, but the level of H_2_O_2_ decreased more slowly and remained at appreciable levels even after 48 h (Figure 1A). Specifically, PAA decreased sharply within the first 3 h (from ~0.0307% to ~0.0141%) and was close to 0% by ~6–12 h, indicating efficient catalytic decomposition under these conditions. In contrast, H_2_O_2_ decreased from ~0.0226% to ~0.0086% after 6 h, but then remained at ~0.006 to 0.007% from 24 to 48 h.

To determine whether this persistent peroxide could be eliminated, we added 20 g/L glucose into the same HEPES-free Fe-EDTA decomposition medium. Interestingly, glucose markedly accelerated peroxide removal during PAA decomposition, and both compounds decreased rapidly during the first 6 h (Figure 1B). PAA declined from ~0.0305% to ~0.0100% by 3 h and continued to decrease thereafter, reaching ~0.0049% at 6 h, ~0.0028% at 9 h, ~0.0007% at 12 h, and ~0.00009% at 24 h. In parallel, H_2_O_2_ decreased from ~0.0227% to ~0.0104% at 6 to 9 h, and then declined more slowly to the LOQ level at 48 h (Figure 1B). Thus, Fe-catalyzed decomposition of PAA in the absence of HEPES was inhibited by the delayed breakdown of H_2_O_2_, but the addition of glucose enabled more complete decomposition of H_2_O_2_.

### 3.2. Effect of Peptone on Glucose-Accelerated Degradation of Peroxides During Fe-EDTA-Mediated Decomposition of PAA

**Figure 2:**
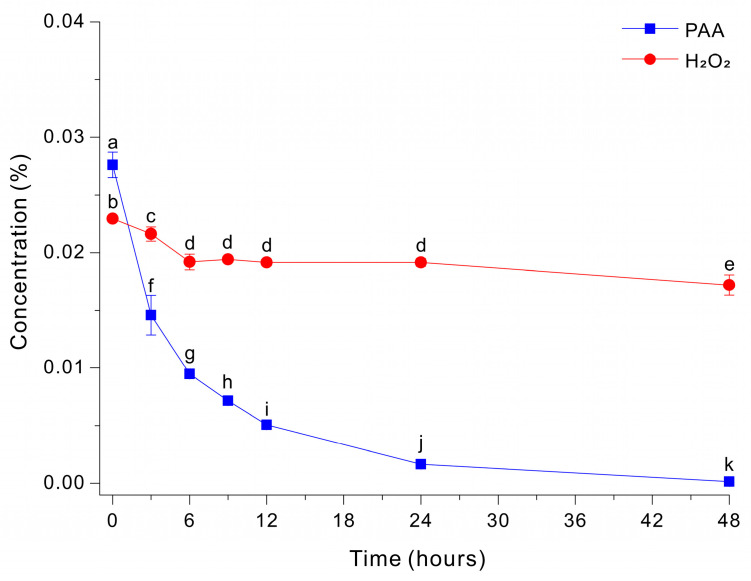
Decomposition of PAA and H_2_O_2_ by a Fe-EDTA-mediated reaction in a HEPES-free 0.03% PAA solution with 20 g/L glucose and 5 g/L peptone. The data were analyzed using one-way ANOVA, followed by Duncan’s test (*p* < 0.05). Lowercase letters indicate statistically significant differences among groups.

The addition of peptone (5 g/L) to the HEPES-free Fe-EDTA-mediated decomposition medium at pH 7 led to slower decomposition of PAA and incomplete removal of peroxide species (Figure 2). Specifically, PAA decreased rapidly during the initial phase, dropping from ~0.0276% to ~0.0146% by ~3 h, and this was followed by a slower decline to ~0.0016% at 48 h. In contrast, H_2_O_2_ remained essentially unchanged at ~0.0172 to 0.0229% throughout the entire monitoring period. These results indicate that peptone suppressed the decomposition of peroxide under these conditions.

### 3.3. Degradation of PAA and H_2_O_2_ by Na_2_S_2_O_4_

**Figure 3:**
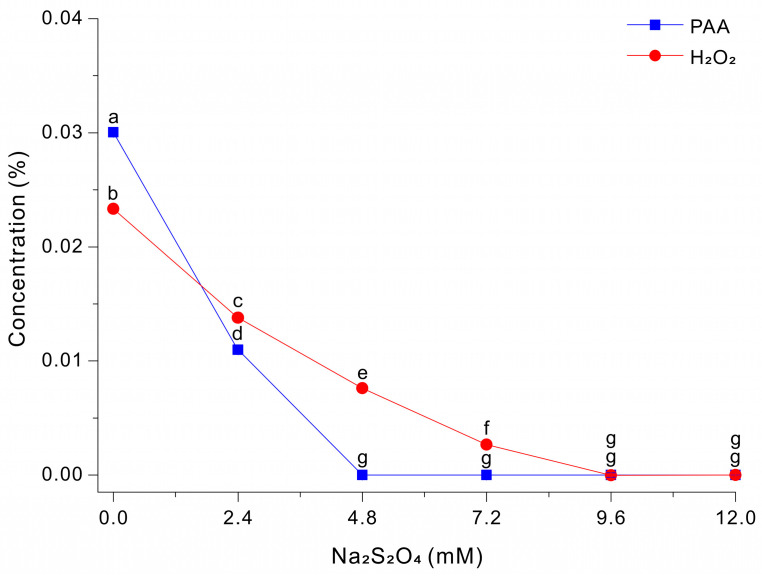
Decomposition of PAA and H_2_O_2_ after treatment with different concentrations of Na_2_S_2_O_4_ in a 0.03% PAA solution for 5 s. The data were analyzed using one-way ANOVA, followed by Duncan’s test (*p* < 0.05). Lowercase letters indicate statistically significant differences among groups.

We then examined the effect of Na_2_S_2_O_4_ on the degradation of PAA and H_2_O_2_ at pH 3. Thus, we treated a 0.03% PAA solution with different concentrations of Na_2_S_2_O_4_ and then measured the residual levels of PAA and H_2_O_2_ after 5 s (Figure 3). The results show that PAA decreased sharply as the Na_2_S_2_O_4_ concentration increased. The PAA level was ~0.0300% at 0 mM Na_2_S_2_O_4,_ ~0.0110% at 2.4 mM Na_2_S_2_O_4,_ and nearly 0 mM at 4.8 to 12 mM Na_2_S_2_O_4_. In contrast, the level of H_2_O_2_ had a more gradual decline as the level of Na_2_S_2_O_4_ increased and only approached 0% at 9.6 to 12.0 mM. These results indicate that Na_2_S_2_O_4_ reacts more readily with PAA-derived oxidants than with H_2_O_2_ under these conditions, and that the Na_2_S_2_O_4_ concentration can be adjusted to selectively quench PAA or to quench PAA and peroxide species.

### 3.4. Na_2_S_2_O_4_-Mediated Rapid Decomposition of PAA Under Various Medium Conditions

When a solution contains peptone, Fe–EDTA-mediated decomposition of PAA is delayed, regardless of the presence of glucose and HEPES (Figure 2). Therefore, the rapid Na_2_S_2_O_4_-mediated quenching of PAA in the presence of peptone was examined in this study. In addition, we evaluated whether Na_2_S_2_O_4_ could completely quench a 0.03% PAA solution under various pH conditions (3, 5, 7, 9, and 11), as well as in the absence of HEPES buffer or EDTA. As a result, across all tested medium conditions—including the presence of organic nitrogen (peptone), a wide pH range (3–11), and conditions with or without HEPES buffer or EDTA—the peroxides in the 0.03% PAA solution were completely quenched within 5 s when Na_2_S_2_O_4_ was applied at a concentration of 9.6 mM (Table 1). Collectively, these findings indicate that Na_2_S_2_O_4_-mediated quenching of PAA is extremely rapid and largely independent of medium composition.

### 3.5. Growth and Paramylon Production of Cells in PAA-Treated Medium

Na_2_S_2_O_4_ is a highly potent reducing agent that is lethal to cells, even at very low concentrations. Our preliminary experiments with *E. gracilis* indicated no cell motility when the residual Na_2_S_2_O_4_ concentration was 0.032 mM or higher. Therefore, considering the relatively high tolerance of *E. gracilis* to H_2_O_2_, we adjusted the concentration of Na_2_S_2_O_4_ to 7.2 mM, a concentration that completely quenches PAA oxidants. Although some residual H_2_O_2_ remained in the medium, this H_2_O_2_ and the small amount of residual Na_2_S_2_O_4_ are unlikely to inhibit cell growth (Figure 3).

**Figure 4:**
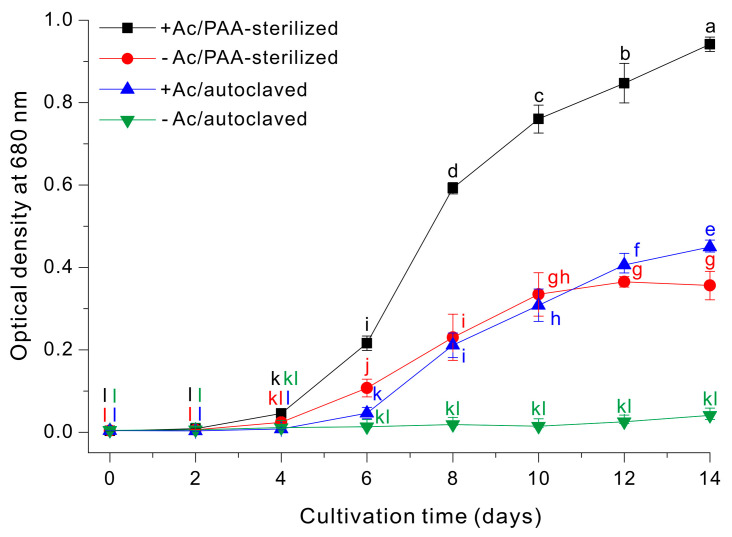
Growth of *E. gracilis* cells in a medium with or without sodium acetate that was prepared by PAA-sterilization or autoclaving. The data were analyzed using one-way ANOVA, followed by Duncan’s test (*p* < 0.05). Lowercase letters indicate statistically significant differences among groups.

We then examined the growth profiles (OD_680nm_) of *E. gracilis* using four different growth media: (i) 10 g/L sodium acetate with autoclaving (+Ac/autoclaved); (ii) 10 g/L sodium acetate with PAA-sterilization (+Ac/PAA-sterilized); (iii) autoclaving alone (−Ac/autoclaved); and (iv) PAA-sterilization alone (−Ac/PAA-sterilized). Growth was clearly optimal in the +Ac/PAA-sterilized medium, intermediate in the –Ac/PAA-sterilized medium and the +Ac/autoclaved medium, and only negligible in the –Ac/autoclaved medium (Figure 4).

**Figure 5:**
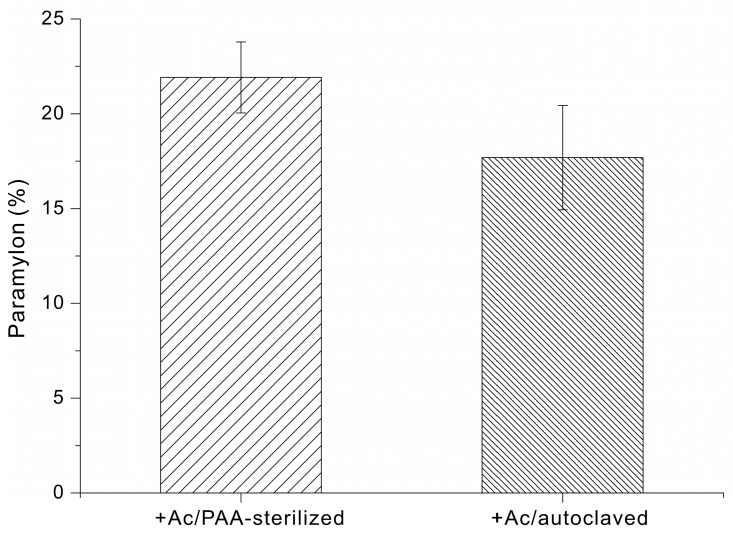
Paramylon content (% of dry cell weight) of *E. gracilis* grown in medium supplemented with sodium acetate and sterilized via PAA treatment or autoclaving.

Finally, we determined the effect of different types of growth media on paramylon production by *E. gracilis*. When cells were grown in +Ac/PAA-sterilized medium, for 14 days the paramylon content was 22 ± 2% of dry cell weight. When cells were grown in +Ac/autoclaved medium, the paramylon content was 18 ± 3% of dry cell weight (Figure 5).

## 4. Discussion

The Fe-EDTA-mediated sterilization of PAA is an attractive approach to sterilization because it uses catalytic redox cycling to decompose PAA-derived oxidants, thereby enabling sterilization of heat-labile components and polymer-based cultivation equipment. Notably, our previous study showed that a 0.03% PAA solution exhibits broad-spectrum biocidal activity, effectively inactivating most microorganisms, including bacteria, fungi, and spores, and that it can be completely decomposed within 12 h under optimized conditions [15,16,18,19]. However, because this approach relies on Fe-catalyzed reaction pathways, its efficacy can be decreased by medium chemistry, such as high pH, HEPES, organic nitrogen sources (peptone), and inorganic nitrogen salts (NaNO_3_). The HEPES-free Fe-EDTA-mediated decomposition process described herein fully decomposed PAA, but this required a reaction time of approximately 24 h. Moreover, neutralization of the co-existing H_2_O_2_ was slow, in that ~0.007% H_2_O_2_ remained after 48 h (Figure 1A). The Fe-EDTA catalyzed reaction of PAA therefore occurred in two stages: a rapid degradation of PAA and a slow and incomplete degradation of H_2_O_2_. It is possible that the absence of HEPES delayed neutralization, because HEPES is not only a pH buffer but can also maintain reaction conditions that support sustained iron redox cycling and efficient decomposition of peroxides [28]. However, without HEPES, the pH of the reaction solution will change, and even a modest change of pH could suppress the decay of peroxides, particularly H_2_O_2_. In addition, under HEPES-free conditions, the speciation and availability of catalytically active iron may be lower. Fe that is sequestered by competing ligands may undergo hydrolysis or precipitation or form less reactive complexes, thus decreasing the concentration of active catalyst.

Glucose exists partly in an open-chain form in solution and contains an aldehyde group (reducing group), allowing it to donate electrons (or hydrogen) and reduce other compounds [29,30]. Therefore, we examined the effect of glucose supplementation on Fe-catalyzed neutralization of PAA under HEPES-free conditions. During HEPES-free Fe-EDTA-mediated decomposition, the kinetics of PAA decay was similar in the absence and presence of glucose (Figure 1A,B). However, glucose supplementation substantially increased the decomposition of H_2_O_2_, and the H_2_O_2_ level was nearly 0% at 24 to 48 h (Figure 1B). This is likely because the Fe-EDTA-mediated reaction alone rapidly decomposes PAA, but complete removal of H_2_O_2_ is rate-limited by Fe redox cycling; thus, glucose apparently acts as an auxiliary electron donor that sustains the Fe(III)/Fe(II) cycle and promotes H_2_O_2_ decomposition [29,30]. Specifically, we can suggest two possible mechanisms by which glucose promotes the decomposition of residual H_2_O_2_: (i) it functions as an electron donor that sustains Fe(III)/Fe(II) redox cycling and thereby prolongs catalyst-driven peroxide turnover and/or (ii) it reacts with radicals or other oxidative intermediates to propagate chain reactions that accelerate oxidant consumption. A previous study described a sterilization–neutralization strategy in which a microalgal growth medium was sterilized with 0.02% Ca(ClO)_2_ and then fully neutralized with an MnCl_2_-Na_2_EDTA catalyst system within 8 h. In this previous study, glucose supplementation synergistically enhanced the MnCl_2_-Na_2_EDTA-catalyzed decay of oxidants and led to complete neutralization [17].

In the present study, we examined the effect of peptone on the Fe-catalyzed neutralization reaction. When we added peptone (5 g/L) to the glucose-supplemented Fe-EDTA solution, PAA decomposition continued gradually, but the H_2_O_2_ level remained essentially unchanged (~0.017–0.023%) for 48 h (Figure 2). In preliminary experiments in which glycine or glutamic acid was used in place of peptone, no inhibition of PAA or H_2_O_2_ decomposition was observed; rather, peroxide degradation proceeded more rapidly than in the nitrogen-free control. This behavior can be attributed to the fact that single amino acids exhibit weak and reversible coordination with iron and possess negligible radical-scavenging capacity, thereby preserving redox-active Fe^2+^/Fe^3+^ species and sustaining Fenton-type reactions that promote peroxide decomposition. The reduced decomposition of peroxides, particularly H_2_O_2_, in the presence of peptone can be explained by several mechanisms. First, peptone is a complex mixture containing peptide-bound residues as well as aromatic and sulfur-containing moieties, which can sequester iron ions, scavenge reactive radicals, and disrupt iron redox cycling [31,32]. Second, peptone may contain metal-binding ligands (e.g., histidine, cysteine, and carboxylate-bearing residues) that alter Fe speciation and/or decrease the reactivity of EDTA-bound Fe, thereby decreasing the fraction of catalytically effective and accessible Fe species [33]. Third, diverse organic constituents in peptone may scavenge radicals or other reactive intermediates, leading to decreases in chain propagation and activity of radical-mediated pathways that contribute to the decomposition of H_2_O_2_ [34]. Fourth, peptone may compete with peroxide species for oxidative reactions so that, despite the decomposition of PAA, the catalytic turnover required for sustained H_2_O_2_ decomposition—particularly the regeneration of Fe(II) from Fe(III)—cannot proceed efficiently [35]. Collectively, these findings demonstrate that complex organic nitrogen compounds can inhibit metal-catalyzed neutralization. This emphasizes that medium composition, especially supplementation by organic nitrogen and other complex organics, must be considered when designing a robust sterilization–neutralization process.

Our results indicate that Fe-EDTA-mediated decomposition rapidly consumes PAA, even in the absence of HEPES; however, a truly peroxide-free endpoint depends on elimination of the residual peroxide pool. Glucose supplementation can facilitate the complete removal of H_2_O_2_ (Figure 1A), but the presence of complex organic nitrogen sources, such as peptone, can negate this effect (Figure 2). In addition, neutralization of a PAA solution used for sterilization of microbial media via metal-catalyzed pathways, such as Fe-EDTA-mediated decomposition, requires at least 6 h under optimized conditions [15,16,18,19], making this approach unsuitable for many operating environments and media. This motivated us to develop an alternative PAA neutralization technology that does not have these limitations.

Sodium dithionite (Na_2_S_2_O_4_; also termed sodium hydrosulfite) is a sulfur(IV)-based inorganic salt that is widely used as a strong reducing agent in aqueous systems [25,36]. In water, dithionite functions as an efficient electron donor that is readily oxidized, and this property is responsible for its rapid reductive deoxygenation and chemical quenching of many oxidants. The results of the present study showed that Na_2_S_2_O_4_ provided a fundamentally different mode of neutralization than metal-catalyzed pathways. In particular, Na_2_S_2_O_4_ acts directly as a stoichiometric reductant that rapidly consumes oxidants present in a PAA solution, does not rely on buffers or pH, is unaffected by the presence of peptone, and does not require a prolonged reaction time. Importantly, Na_2_S_2_O_4_ can be used in acidic environments, and maintaining a low pH during the quenching/neutralization helps ensure robust sterilization [23]. By contrast, Fe-EDTA-mediated decomposition typically requires a pH near 7.0 to efficiently degrade peroxide species, and this can decrease the benefit of the strong biocidal activity of PAA at low pH.

Complete neutralization of the 0.03% PAA solution occurred within 5 s after the addition of 9.6 mM Na_2_S_2_O_4_ (Figure 3). Interestingly, Na_2_S_2_O_4_ also had clear reactivity against the two oxidant pools (PAA-derived oxidants and H_2_O_2_). Specifically, PAA-derived oxidants were quenched efficiently at a relatively low Na_2_S_2_O_4_ concentration, with near complete removal by ~4.8 mM Na_2_S_2_O_4_; near-complete quenching of H_2_O_2_ required a higher concentration of Na_2_S_2_O_4_ (~9.6–12.0 mM) (Figure 3). This difference provides a practical lever for tuning the neutralization endpoint based on Na_2_S_2_O_4_ concentration. From a bioprocess perspective, this selectivity is important because Na_2_S_2_O_4_ is a neutralizing reductant that can be cytotoxic if it remains at appreciable levels after treatment. For microorganisms that have a comparatively high tolerance to H_2_O_2_, such as *E. gracilis*, it may be advantageous to exploit the differential reactivity of Na_2_S_2_O_4_ by selecting a dose that fully quenches PAA while allowing a residual level of H_2_O_2_ to remain. If the target strain has greater tolerance to H_2_O_2_ than PAA-derived oxidants, this strategy could reduce the risk of reductant-associated toxicity by Na_2_S_2_O_4_ and maintain a mild residual oxidative background that may contribute to antimicrobial protection during early cultivation.

In this study, we leveraged the comparatively high H_2_O_2_ tolerance of *E. gracilis* to define an operational setpoint of 7.2 mM Na_2_S_2_O_4_ [37]. This concentration ensured complete quenching of PAA, although some H_2_O_2_ remained (Figure 3). This approach greatly decreased the amount of residual dithionite so there was no inhibition of cell growth. A growth medium prepared using this strategy outperformed conventional autoclave sterilization (Figure 4). Under acetate-supplemented conditions (+Ac, 10 g/L), PAA sterilization followed by Na_2_S_2_O_4_ quenching consistently led to greater cell growth than the autoclaved control. PAA sterilization likely has an additional advantage over thermal sterilization. Autoclaving can alter medium chemistry via degradation or modification of labile constituents and may generate growth-inhibitory byproducts (e.g., melanoidins and other browning-related reaction products) that decrease the quality of the growth medium [14,15,16,18,38]. In contrast, PAA-sterilization achieves decontamination without high temperature and pressure. As a result, PAA-sterilization enables greater accumulation of biomass (Figure 4). In addition, under acetate-free conditions (−Ac), the autoclaved medium had very little cell growth over 14 days, whereas the medium sterilized by PAA followed by Na_2_S_2_O_4_ quenching supported substantial growth (Figure 4). This outcome is plausibly attributable to the acetic acid generated during PAA decomposition/neutralization, which *Euglena* can use as a carbon source. In agreement, previous studies also showed that sterilization with PAA followed by neutralization led to greater growth of target organisms [15,16,18,19].

In addition to sterilization-dependent differences in biomass accumulation, the paramylon content was also greater following the PAA sterilization and Na_2_S_2_O_4_ quenching process (22 ± 2%) than after autoclaving (18 ± 3%) (Figure 5). The increased paramylon content observed following PAA sterilization and Na_2_S_2_O_4_ quenching may be attributed to improved preservation of medium integrity under non-thermal conditions. Although the underlying metabolic pathways were not directly investigated in this study, it is conceivable that avoiding heat limits glucose degradation and the formation of inhibitory byproducts, thereby maintaining more effective carbon availability for storage polysaccharide biosynthesis. In contrast, autoclaving could generate heat-derived compounds that impose metabolic stress and potentially redirect cellular resources away from paramylon accumulation.

## 5. Conclusions

This study demonstrated that Na_2_S_2_O_4_ provided a robust, rapid, and wash-free method for quenching PAA-sterilized media and addressed the practical limitations of metal-catalyzed PAA decomposition/neutralization. Fe-EDTA-mediated decomposition often requires a near-neutral pH and a buffer and can be inhibited by complex organic constituents that lead to slow or incomplete removal of oxidants. In contrast, Na_2_S_2_O_4_ acted as a direct reductant that rapidly quenched PAA-derived oxidants without requiring pH adjustment or a buffer and was not sensitive to the presence of nitrogen compounds. In addition, Na_2_S_2_O_4_ exhibited clear differential reactivity, in that it degraded PAA at a relatively low concentration (~4.8 mM), but a higher concentration was needed to degrade H_2_O_2_ (~9.6–12.0 mM). This selectivity enabled the establishment of an operational window (~4.8–7.2 mM) in which PAA could be fully quenched while a controllable residual amount of H_2_O_2_ remained. Considering the strong cytotoxicity of residual Na_2_S_2_O_4_ (≥0.032 mM) and the relatively high tolerance of H_2_O_2_ by *E. gracilis*, we used 7.2 mM Na_2_S_2_O_4_ for quenching 0.03% PAA-sterilized media. Our optimized PAA-Na_2_S_2_O_4_ sterilization–neutralization process for preparing cultivation media for *E. gracilis* led to greater biomass and greater production of paramylon than conventional autoclaving.

## Figures and Tables

**Table 1:** Na_2_S_2_O_4_-mediated quenching of a PAA solution under various medium conditions.

	Peptone		pH Condition					EDTA		HEPES	
Condition	0 g/L	5 g/L	3	5	7	9	11	0 µM	96 µM	0 mM	10 mM
Complete quenching of peroxides in 0.03% PAA	-	-	-	-	-	-	-	-	-	-	-

- indicates that the peroxides present in the 0.03% PAA solution (PAA and H_2_O_2_) were completely quenched within 5 s after reaction with 9.6 mM Na_2_S_2_O_4_.

## Data Availability

The original contributions presented in this study are included in the article. Further inquiries can be directed to the corresponding author.

## References

[1] Kottuparambil S., Thankamony R.L., Agusti S. (2019). *Euglena* as a potential natural source of value-added metabolites. A review. Algal Res..

[2] Gissibl A., Sun A., Care A., Nevalainen H., Sunna A. (2019). Bioproducts from *Euglena gracilis*: Synthesis and applications. Front. Bioeng. Biotechnol..

[3] Zhu L., Liu M., Wang Y., Zhu Z., Zhao X. (2024). *Euglena gracilis* protein: Effects of different acidic and alkaline environments on structural characteristics and functional properties. Foods.

[4] Takeyama H., Kanamaru A., Yoshino Y., Kakuta H., Kawamura Y., Matsunaga T. (1997). Production of antioxidant vitamins, β-carotene, vitamin C, and vitamin E, by two-step culture of *Euglena gracilis* Z. Biotechnol. Bioeng..

[5] Hayashi M., Toda K., Kitaoka S. (1993). Enriching *Euglena* with unsaturated fatty acids. Biosci. Biotechnol. Biochem..

[6] Chaudhuri D., Ghate N.B., Deb S., Panja S., Sarkar R., Rout J., Mandal N. (2014). Assessment of the phytochemical constituents and antioxidant activity of a bloom forming microalgae *Euglena* tuba. Biol. Res..

[7] Xie W., Li X., Xu H., Chen F., Cheng K.-W., Liu H., Liu B. (2023). Optimization of heterotrophic culture conditions for the microalgae *Euglena gracilis* to produce proteins. Mar. Drugs.

[8] Mahapatra D.M., Chanakya H., Ramachandra T. (2013). *Euglena* sp. as a suitable source of lipids for potential use as biofuel and sustainable wastewater treatment. J. Appl. Phycol..

[9] Kim S., Im H., Yu J., Kim K., Kim M., Lee T. (2023). Biofuel production from *Euglena*: Current status and techno-economic perspectives. Bioresour. Technol..

[10] Perez-Garcia O., Bashan Y., Esther Puente M. (2011). Organic carbon supplementation of sterilized municipal wastewater is essential for heterotrophic growth and removing ammonium by the microalga *Chlorella Vulgaris* 1. J. Phycol..

[11] Mubarak M.T., Ozsahin I., Ozsahin D.U. Evaluation of sterilization methods for medical devices. Proceedings of the 2019 Advances in Science and Engineering Technology International Conferences (ASET).

[12] Cardoso J.C., Imthurn A.C.P. (2018). Easy and efficient chemical sterilization of the culture medium for in vitro growth of *gerbera* using chlorine dioxide (ClO_2_). Ornam. Hortic..

[13] Ball E. (1953). Hydrolysis of sucrose by autoclaving media, a neglected aspect in the technique of culture of plant tissues. Bull. Torrey Bot. Club.

[14] Lim H.-J., Kang M.-S., Kwon J.-H. (2025). Sterilization of culture systems using oxidation–reduction cycles of iodine for efficient, cost-effective, and sustainable bioprocessing: Application for *Euglena* cultivation and production of paramylon. J. Appl. Phycol..

[15] Chang-Ho C., Do-Wook W., Jong-Hee K. Development of Cultivation Process Using Decomposition of PAA for Cost-efficient Omega-3 Production by *Aurantiochytrium* sp. Proceedings of the 2017 KSBB Fall Meeting and International Symposium (Abstract Book), BEXCO.

[16] Cho C.-H., Shin W.-S., Woo D.-W., Kwon J.-H. (2018). Growth medium sterilization using decomposition of peracetic acid for more cost-efficient production of omega-3 fatty acids by *Aurantiochytrium*. Bioprocess Biosyst. Eng..

[17] Jeong S.-H., Kim W., Kwon J.-H. (2024). Development of a new sterilization method for microalgae media using calcium hypochlorite as the sterilant. Bioprocess Biosyst. Eng..

[18] Lee G.-I., Shin W.-S., Jung S.M., Kim W., Lee C., Kwon J.-H. (2020). Effects of soybean curd wastewater on growth and DHA production in *Aurantiochytrium* sp. LWT.

[19] Sung M.-G., Lee H., Nam K., Rexroth S., Rögner M., Kwon J.-H., Yang J.-W. (2015). A simple method for decomposition of peracetic acid in a microalgal cultivation system. Bioprocess Biosyst. Eng..

[20] Jeong Y., Choi W.-Y., Park A., Lee Y.-J., Lee Y., Park G.-H., Lee S.-J., Lee W.-K., Ryu Y.-K., Kang D.-H. (2021). Marine cyanobacterium *Spirulina maxima* as an alternate to the animal cell culture medium supplement. Sci. Rep..

[21] Wolf D., Dao T., Scott H., Lavy T. (1989). Influence of Sterilization Methods on Selected Soil Microbiological, Physical, and Chemical Properties.

[22] Kheirabadi S., Sheikhi A. (2022). Recent advances and challenges in recycling and reusing biomedical materials. Curr. Opin. Green Sustain. Chem..

[23] Kitis M. (2004). Disinfection of wastewater with peracetic acid: A review. Environ. Int..

[24] Lambeth D.O., Palmer G. (1973). The kinetics and mechanism of reduction of electron transfer proteins and other compounds of biological interest by dithionite. J. Biol. Chem..

[25] Makarov S.V. (2001). Recent trends in the chemistry of sulfur-containing reducing agents. Russ. Chem. Rev..

[26] Xiao J., Wang M., Pang Z., Dai L., Lu J., Zou J. (2019). Simultaneous spectrophotometric determination of peracetic acid and the coexistent hydrogen peroxide using potassium iodide as the indicator. Anal. Methods.

[27] Kim K., Kang J., Seo H., Kim S., Kim D.Y., Park Y., Yu J., Lee T. (2024). A novel screening strategy utilizing aniline blue and calcofluor white to develop paramylon-rich mutants of *Euglena gracilis*. Algal Res..

[28] Park S., Gakh O., O’Neill H.A., Mangravita A., Nichol H., Ferreira G.C., Isaya G. (2003). Yeast frataxin sequentially chaperones and stores iron by coupling protein assembly with iron oxidation. J. Biol. Chem..

[29] Madani M., Hosny S., Alshangiti D.M., Nady N., Alkhursani S.A., Alkhaldi H., Al-Gahtany S.A., Ghobashy M.M., Gaber G.A. (2022). Green synthesis of nanoparticles for varied applications: Green renewable resources and energy-efficient synthetic routes. Nanotechnol. Rev..

[30] Saikhao L., Setthayanond J., Karpkird T., Suwanruji P. Comparison of sodium dithionite and glucose as a reducing agent for natural indigo dyeing on cotton fabrics. Proceedings of the MATEC Web of Conferences.

[31] Abbasiliasi S., Tan J.S., Ibrahim T.A.T., Bashokouh F., Ramakrishnan N.R., Mustafa S., Ariff A.B. (2017). Fermentation factors influencing the production of bacteriocins by lactic acid bacteria: A review. RSC Adv..

[32] Hayek S.A., Gyawali R., Aljaloud S.O., Krastanov A., Ibrahim S.A. (2019). Cultivation media for lactic acid bacteria used in dairy products. J. Dairy Res..

[33] Gaigher B., da Silva E.d.N., Sanches V.L., Milani R.F., Galland F., Cadore S., Grancieri M., Pacheco M.T.B. (2022). Formulations with microencapsulated Fe–peptides improve in vitro bioaccessibility and bioavailability. Curr. Res. Food Sci..

[34] Sun K.-L., Gao M., Wang Y.-Z., Li X.-R., Wang P., Wang B. (2022). Antioxidant peptides from protein hydrolysate of marine red algae *Eucheuma cottonii*: Preparation, identification, and cytoprotective mechanisms on H_2_O_2_ oxidative damaged HUVECs. Front. Microbiol..

[35] Jin S.K., Choi J.S., Choi Y.J., Lee S.-J., Lee S.Y., Hur S.J. (2016). Antioxidant, liver protective and angiotensin I-converting enzyme inhibitory activities of old laying hen hydrolysate in crab meat analogue. Asian-Australas. J. Anim. Sci..

[36] Makarov S.V., Silaghi-Dumitrescu R. (2013). Sodium dithionite and its relatives: Past and present. J. Sulfur Chem..

[37] Nowicka B. (2022). Heavy metal–induced stress in eukaryotic algae—Mechanisms of heavy metal toxicity and tolerance with particular emphasis on oxidative stress in exposed cells and the role of antioxidant response. Environ. Sci. Pollut. Res..

[38] Leitzen S., Vogel M., Steffens M., Zapf T., Müller C.E., Brandl M. (2021). Quantification of degradation products formed during heat sterilization of glucose solutions by LC-MS/MS: Impact of autoclaving temperature and duration on degradation. Pharmaceuticals.

